# Case Report: Changes in Cytokine Kinetics During the Course of Disease in a Japanese Patient With Multisystem Inflammatory Syndrome in Children

**DOI:** 10.3389/fped.2021.702318

**Published:** 2021-07-21

**Authors:** Satoshi Takasago, Aiko Sakai, Masaya Sugiyama, Masashi Mizokami, Hiromichi Hamada, Yukihito Ishizaka, Tohru Miyoshi-Akiyama, Akihiro Matsunaga, Mikako Ueno, Hiroyuki Shichino, Ayumi Mizukami

**Affiliations:** ^1^Department of Pediatrics, Center Hospital of the National Center for Global Health and Medicine, Tokyo, Japan; ^2^Genome Medical Sciences Project, Research Institute, National Center for Global Health and Medicine, Tokyo, Japan; ^3^Department of Pediatrics, Graduate School of Medicine, Chiba University, Chiba, Japan; ^4^Department of Intractable Diseases, Research Institute, National Center for Global Health and Medicine, Tokyo, Japan; ^5^Department of Infectious Diseases, Research Institute, National Center for Global Health and Medicine, Tokyo, Japan

**Keywords:** MIS-C, PIMS-TS, COVID-19, SARS-CoV-2, Kawasaki disease, cytokine, case report

## Abstract

Multisystem inflammatory syndrome in children (MIS-C) is a severe disease that is reportedly linked to coronavirus disease 2019. Affected patients present with gastrointestinal symptoms and cardiovascular dysfunction, in addition to Kawasaki disease-like features, suggesting the potential for overlapping disease mechanisms. Kawasaki disease has been reported among individuals of East Asian ethnicities, whereas there is minimal clinical literature regarding the occurrence of MIS-C among individuals of Asian ethnicities. A few reports thus far have described changes in cytokine kinetics during the course of disease in patients with MIS-C. We followed the temporal cytokine kinetics in a 9-year-old Japanese girl who exhibited a classical trajectory of MIS-C. The patient exhibited right cervical swelling and pain, abdominal pain, vomiting, and lip reddening, which developed 31 days after she was diagnosed with severe acute respiratory syndrome coronavirus-2 infection. The patient was diagnosed with Kawasaki disease on her fifth day of illness; because she fulfilled the criteria for MIS-C, she was also diagnosed with this disease on her fifth day of illness. Her fever rapidly resolved upon administration of intravenous immunoglobulin, aspirin, and prednisolone. On the patient's sixth day of illness, she developed acute myocarditis, which was treated with two diuretics and one vasodilator; the myocarditis ameliorated within a few days. Analyses of temporal kinetics for 71 serum cytokines revealed several patterns of cytokine changes that were consistent with the patient's clinical course of disease. Importantly, there was a clear distinction between cytokines that did and did not decrease rapidly following post-treatment fever resolution. These findings may be useful for the assessment of disease status and selection of therapy in patients with similar symptoms; they may also provide insights for basic and clinical research regarding MIS-C.

## Introduction

Multisystem inflammatory syndrome in children (MIS-C; also known as pediatric inflammatory multisystem syndrome toxic shock) is a condition that has been linked with coronavirus disease 2019 (COVID-19) mainly in Europe and North America since April 2020; it reportedly involves serious complications including Kawasaki disease-like features (1, 2). MIS-C occurs within 2–6 weeks after an acute infection with severe acute respiratory syndrome coronavirus-2 (SARS-CoV-2). Distinct features include an older peak age of onset, compared with patients with classical Kawasaki disease (3); they also include frequent gastrointestinal symptoms and greater incidences of both cardiovascular dysfunction and intensive care management than in patients with classical Kawasaki disease (4). While Kawasaki disease is more prominent among individuals of East Asian ethnicities, MIS-C reportedly occurs more often among individuals of Afro-Caribbean ethnicities (5); to the best of our knowledge, there is minimal clinical literature regarding the occurrence of MIS-C among individuals of Asian ethnicities. Furthermore, a few reports thus far have described changes in cytokine kinetics during the course of MIS-C with sufficient detail to identify cytokine changes in relation to clinical manifestations. We presume that an understanding of these changes may help to elucidate the underlying etiology of MIS-C. Here, we describe a patient whose course of MIS-C followed a trajectory similar to that observed in previously reported patients; additionally, we describe the temporal kinetics of 71 cytokines, which were analyzed using serum from our patient.

## Case Description

A 9-year-old Japanese girl with no notable history of medical problems was diagnosed with SARS-CoV-2 infection on the basis of polymerase chain reaction (PCR) analysis of nasopharyngeal swab sample. Her parents are both Japanese. The grandmother was the first in the family to be infected with SARS-CoV-2. Subsequently, the patient, both parents, and one sibling were positive for SARS-CoV-2 on PCR test. There was no contact with SARS-CoV-2 positive patients at school or other places during the same period. The patient exhibited fever and olfactory dysfunction for 2 days, although her symptoms were mild. She did not require hospitalization and spontaneously recovered during in-home quarantine. At 31 days after the patient had been diagnosed with SARS-CoV-2 infection, she developed fever, swelling and pain in the right cervical region, as well as abdominal pain and vomiting. One day later, she presented to our hospital for outpatient treatment. Although the patient was able to return home for approximately 24 hours, her symptoms subsequently worsened; she required emergency admission on the following day (third day of illness).

Upon admission, the patient exhibited a fever of 40.2°C, spontaneous pain throughout the abdomen, vomiting, and right cervical pain. Physical examination revealed multilocular cervical lymphadenopathy with a maximum diameter of 30 mm, mild redness of the lips and oral cavity. There were no indications of bulbar conjunctival congestion, rash, or distal extremity changes. The patient's blood pressure was 113/70 mmHg (i.e., within the reference range). She had a positive test result for SARS-CoV-2, according to FilmArray (bioMérieux SA, Marcy-l'Étoile, France) analysis of a nasopharyngeal swab. Elevated leukocyte count (9.39 × 10^3^/μL), elevated neutrophil count (8.45 × 10^3^/μL), reduced lymphocyte count (0.47 × 10^3^/μL), elevated C-reactive protein level (10.77 mg/dL), and reduced serum sodium level (131 mEq/L) were noted. Platelet count (234 × 10^3^/μL) was within the reference range. Chest and abdominal X-ray findings were normal.

**Figure 1:**
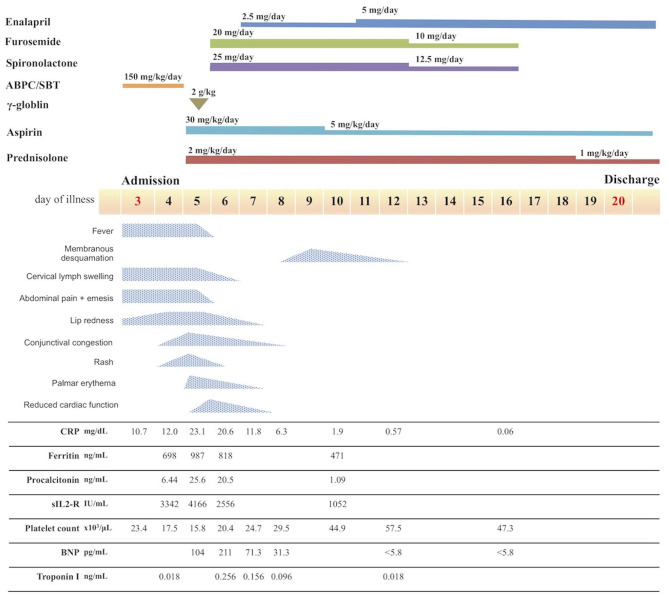
Course of disease after admission. ABPC/SBT, ampicillin/sulbactam; CRP, C-reactive protein; sIL2-R, soluble interleukin-2 receptor; BNP, B-type natriuretic peptide.

The laboratory measures from hospitalization to diagnosis are shown in Table 1; the patient's course of disease following hospitalization is shown in Figure 1. After admission, the patient was diagnosed with purulent cervical lymphadenitis; she then began broad-spectrum antimicrobial treatment with ampicillin/sulbactam (150 mg/kg/day). However, the fever persisted and the patient showed increasing levels of inflammatory reaction markers (e.g., C-reactive protein, erythrocyte sedimentation rate, and procalcitonin). On the patient's fifth day of illness, she developed bilateral bulbar conjunctival congestion, anterior chest erythema, and right palm erythema, in addition to exacerbation of lip reddening; the patient thus fulfilled all six Japanese criteria for Kawasaki disease (6) (Supplementary Figure 1). The patient did not exhibit bacillus Calmette–Guérin inoculation site redness. Furthermore, she exhibited elevated leukocyte count (14.07 × 10^3^/μL), elevated neutrophil count (12.38 × 10^3^/μL), elevated C-reactive protein level (23.05 mg/dL), elevated procalcitonin level (25.56 ng/mL), elevated aspartate aminotransferase level (62 U/L), elevated alanine aminotransferase level (52 U/L), reduced platelet count (158 × 10^3^/μL), prolonged prothrombin time (14.3 s; international normalized ratio: 1.17), elevated fibrinogen level (691 mg/dL), and elevated D-dimer level (4.9 μg/mL) (Table 1). Because the patient fulfilled the WHO preliminary criteria (7), she was diagnosed with MIS-C.

**Table 1:** Blood test results from admission (3rd day of illness) to MIS-C diagnosis (5th day of illness).

	**Day 3**	**Day 4**	**Day 5**	**Reference range**
White blood cell (×10^3^/μL)	9.39	7.63	14.07	4.5–13.5
Neutrophil (×10^3^/μL)	8.45	6.71	12.38	1.8–8.0
Lymphocyte (×10^3^/μL)	0.47	0.53	1.41	1.5–6.5
Hemoglobin (g/dL)	14.0	11.9	12.3	11.5–14.8
Platelets (×10^4^/μL)	23.4	18.4	15.8	15.0–40.0
Albumin (g/dL)	3.9	3.2	3.2	3.5–5.0
Total bilirubin (mg/dL)	0.6	0.5	0.5	0.4–1.5
Aspartate aminotransferase (IU/L)	41	60	62	16–38
Alanine aminotransferase (IU/L)	25	39	52	4.0–25.0
Lactate dehydrogenase (IU/L)	276	288	322	286–606
Creatinine kinase (IU/L)	140	372	198	41–212
Blood urea nitrogen (mg/dL)	13.4	8.0	8.3	8.0–20.0
Creatinine (mg/dL)	0.36	0.35	0.46	0.34–0.51
Sodium (mEq/L)	131	126	130	138–145
Potassium (mEq/L)	3.9	3.2	3.6	3.4–4.7
Chloride (mEq/L)	95	94	94	98–106
C-reactive protein (mg/dL)	10.77	9.57	23.05	<0.15
Erythrocyte sedimentation rate (mm/h)	52	54	N/A	5–15
Ferritin (ng/mL)	N/A	698	987	5–157
Procalcitonin (ng/mL)	N/A	6.44	25.56	<0.05
Prothrombin time (sec)	13.3	14.1	14.3	11.0–13.0
PT-international normalized ratio	1.08	1.15	1.17	0.75–1.15
Fibrinogen (mg/dL)	669	531	691	200–400
D-dimer (μg/mL)	0.9	2.4	4.9	<1.0
B-natriuretic peptide (pg/mL)	N/A	N/A	104.0	<18.4
Troponin I (ng/mL)	N/A	0.018	N/A	<0.026
Soluble interleukin-2 receptor (IU/mL)	N/A	3,342	4,166	122–496

*N/A, not available*.

**Figure 2:**
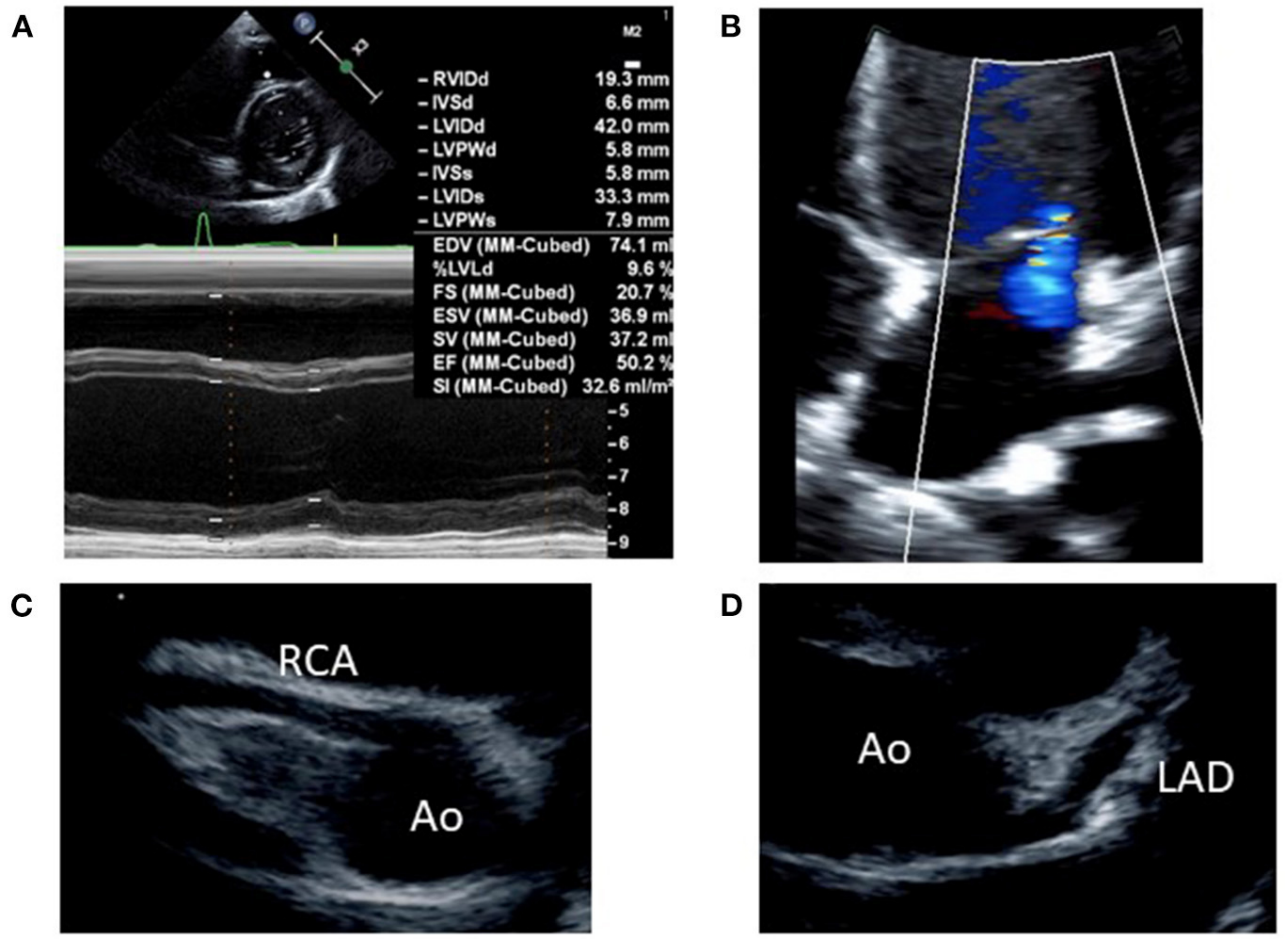
Echocardiography findings on the 6th day of illness: **(A)** M-mode (left ventricular ejection fraction, 50.2%), **(B)** mild mitral regurgitation by 4-chamber view, **(C)** right coronary artery (seg1: 3.0 mm, *Z* score+1.6), *Z* score was calculated by “*Z* score calculator of coronary arterial diameter” (http://raise.umin.jp/zsp/calculator/), **(D)** left coronary artery (seg5: 3.0 mm, *Z* score+0.9, seg6: 2.7 mm, *Z* score+1.3, seg11: 2.6 mm, *Z* score+1.5). Ao, aorta; RCA, right coronary artery; LAD, left anterior descending artery.

Following diagnosis of MIS-C, the patient exhibited normal cardiac function according to echocardiography performed prior to treatment start, with no indications of pericardial effusion. She did not exhibit coronary artery dilation, but demonstrated enhanced coronary artery wall brightness. Furthermore, she had no unusual electrocardiographic findings. We expected that the patient was at considerable risk of coronary aneurysm, on the basis of previous findings by Kobayashi et al. (8, 9). Accordingly, we administered intravenous immunoglobulin (IVIG; 2 g/kg) and aspirin (ASA; 30 mg/kg/day), which constitute standard treatment for Kawasaki disease, beginning on the 5th day of illness; we also began administration of prednisolone (PSL; 2 mg/kg/day) at that time. The patient's treatment response was favorable, such that the fever resolved within 12 hours of treatment initiation; the patient's other symptoms showed progressive amelioration. Furthermore, the serum levels of inflammation markers (e.g., C-reactive protein, procalcitonin, ferritin, and soluble interleukin-2 receptor; Figure 1) also decreased relatively rapidly from a peak on the 5th day of illness. On the 6th day of illness, however, she exhibited reduced left ventricular systolic function (ejection fraction 50.2%) and enhanced left ventricular end-diastolic diameter (Figure 2A), as well as pericardial effusion, mild mitral regurgitation (Figure 2B), and mild tricuspid regurgitation. No coronary artery dilation was evident (Figures 2C,D). Moreover, she exhibited ST-T elevations (Supplementary Figure 2) and was diagnosed with acute myocarditis, following observations of elevated serum troponin I level (0.256 ng/mL) and elevated blood B-type natriuretic peptide level (211 pg/mL) in blood tests. There were no symptoms of heart failure, and her blood pressure was 100/50 mmHg. Urine output was maintained. Chest X-ray showed an increased cardiothoracic ratio (55%), but no pleural effusion. Treatment with diuretics (furosemide and spironolactone) was initiated that day; treatment with a vasodilator (enalapril) was initiated on the following day. The patient's cardiac function and ST-T elevations rapidly improved by the 9th day of illness. Her B-type natriuretic peptide level and serum troponin I level were within the respective reference ranges at the 12th day of illness. On the 8th day of illness, she exhibited membranous desquamation from the nail-skin junction of the bilateral toes, suggestive of recovery. No symptom recurrence was noted; inflammatory reaction markers (e.g., C-reactive protein; Figure 1) also decreased relatively rapidly. The ASA dose was reduced to 5 mg/kg/day on the 10th day of illness, the PSL dose was tapered to 1 mg/kg/day on the 19th day of illness, and the patient was discharged on the 20th day of illness. The PSL dose was reduced to 0.5 mg/kg/day at an outpatient visit on the 24th day of illness; PSL treatment was discontinued on the 29th day of illness. The patient continued ASA until 1.5 months after discharge, then discontinued the medication.

The patient was re-tested for SARS-CoV-2 infection on the 11th day of illness by PCR analysis of a nasopharyngeal swab sample; the test result was negative. Moreover, a custom enzyme-linked immunosorbent assay performed with serum collected upon admission, using SARS-CoV-2 nucleocapsid and spike protein purified in-house as the antigen, showed a positive result (nucleocapsid: OD_450_ = 0.447; positive threshold, ≥ 0.211, spike: OD_450_ = 0.484; positive threshold, ≥ 0.155); this confirmed the presence of serum antibodies to SARS-CoV-2.

After completion of treatment, we evaluated temporal changes in the patient's cytokine kinetics, using archived serum samples collected intermittently from the second day to the 19th day of illness. We performed comprehensive assays for cytokines using the BioPlex 3D system (Bio-Rad Laboratories Inc., Hercules, CA, USA) and the BioPlex Human cytokine48/chemokine40 screening panel (Bio-Rad Laboratories Inc.). We also performed chemiluminescence enzyme immunoassay analyses using an HISCL-5000 (Sysmex Asia Pacific Pte Ltd., Singapore) and ELISA assay (R&D systems, Inc.). We investigated changes in these 71 markers during the patient's course of disease (Supplementary Figure 3).

**Figure 3:**
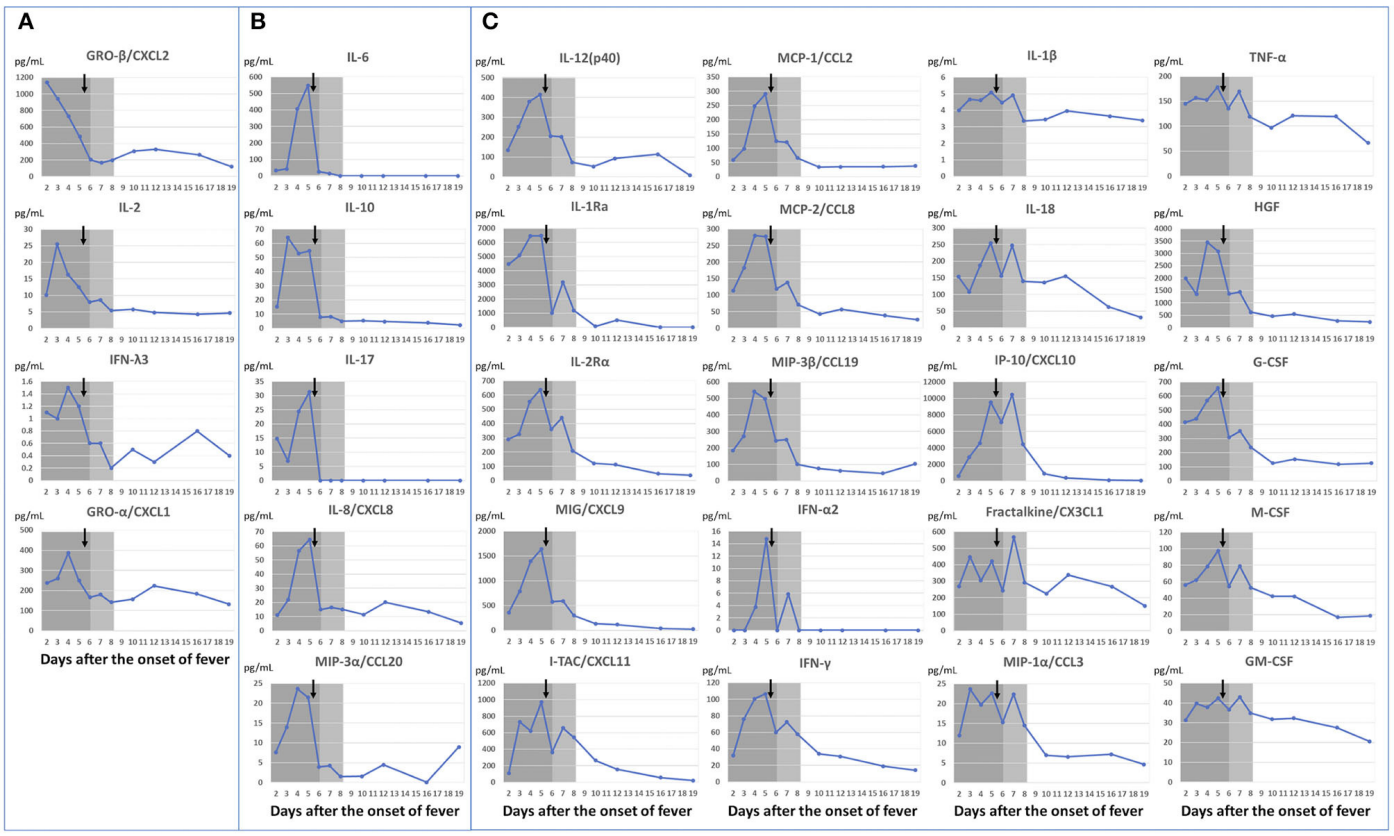
Dynamic changes in cytokines according to changes in clinical symptoms. Febrile period shown in dark gray; duration of myocarditis shown in light gray. ↓ indicates IVIG+PSL+ASA start timing. Dots represent blood draw timings. **(A)** Cytokines with peak titers before treatment initiation. **(B)** Cytokines with rapid reductions following fever resolution. **(C)** Cytokines that persisted at high titers for an extended period following fever resolution, which then decreased with amelioration of myocarditis. ASA, aspirin; IVIG, intravenous immunoglobulin; PSL, prednisolone; IL, interleukin; MIP, macrophage inflammatory protein; IFN, interferon; MCP, monocyte chemoattractant protein; MIG, monokine induced by interferon-γ; IP, interferon-γ-induced protein; TNF, tumor necrosis factor; HGF, hepatocyte growth factor; G-CSF, granulocyte colony-stimulating factor; M-CSF, macrophage colony-stimulating factor; GM-CSF, granulocyte-macrophage colony-stimulating factor.

Cytokine changes were striking, such that temporal cytokine kinetics exhibited five patterns: (1) a peak before treatment initiation on the 5th day of illness (Figure 3A: interleukin (IL)-2, interferon-λ3 et al.); (2) elevation until the 5th day of illness, followed by rapid reduction on the 6th day of illness after treatment initiation and concomitant fever resolution (Figure 3B: IL-6, IL-10, IL-17, IL-8, CCL20); (3) persistence of high titers for several days after fever resolution, followed by a reduction in titers after resolution of myocarditis (Figure 3C: IL-1 receptor antagonist (Ra), CXCL9, CXCL11, CCL2, interferon-γ, IL-1β, IL-18, CXCL10, and tumor necrosis factor-α et al.); (4) low titers in the acute phase, followed by elevation (CCL17 and SCGF-β); and (5) no change [CCL11, CCL15, CCL23, IL-3, IL-4, IL-5, IL-12(p70)]. Elevations of the proliferative/hematopoietic factors (hepatocyte growth factor, granulocyte colony-stimulating factor, macrophage colony-stimulating factor, and granulocyte-macrophage colony-stimulating factor) were also observed in the presence of inflammation.

The patient's parents provided informed consent for publication of this report, including the publication of photographs of the patient's face and body. The patient also provided informed assent. This clinical analysis was performed in accordance with the Declaration of Helsinki and the Ethical Guidelines of the Ministry of Health, Labor and Welfare of Japan; it was approved by the Research Ethics Committees of the National Center for Global Health and Medicine.

## Discussion

This patient exhibited MIS-C involving a spectrum of symptoms along with elevated inflammatory responses within a few days of fever onset. The response to treatment comprising IVIG, ASA, and PSL was good; her inflammatory responses rapidly reduced with fever resolution. The patient's current medical history, symptoms (fever, abdominal pain, nausea, and manifestations of Kawasaki disease), and responses to treatment were also consistent with reports from Europe and the United States (1, 2, 4). Although it is difficult to clearly differentiate MIS-C from Kawasaki shock syndrome, the clinical picture of this case is closer to that of MIS-C because of the obvious preceding SARS-CoV-2 infection, the older age, the strong abdominal pain and vomiting, and the thrombocytopenia and lymphopenia (10). This patient's cardiac function was reduced after fever resolution and required cardiovascular intervention. Based on our clinical experience, we assumed that the effects of vasculitis inflammation on myocardial tissue were delayed. Because cardiac function, vascular permeability, and volume overload due to treatment would all contribute to the patient's disease state, we presumed that reduced cardiac function would occur after the initiation of treatment for MIS-C. In a previous report regarding Kawasaki disease shock syndrome (10), which comprises vasculitis due to hypercytokinemia (similar to MIS-C), hypotension occurred either during or after IVIG administration in three of six patients.

Feldstein et al. (1) reported the findings of a series of 186 patients diagnosed with MIS-C in 26 states within the United States; importantly, Kawasaki disease-like features (e.g., conjunctival congestion, rash, and coronary aneurysm formation) were observed in 74 patients (40%). Furthermore, Toubiana et al. (4) reported that 21 patients with MIS-C had presented with Kawasaki disease-like symptoms and were hospitalized; the median onset age was 7.9 years (3.7–16.6 years), which is higher than the peak age for Kawasaki disease (3). Moreover, all 21 patients had gastrointestinal symptoms such as vomiting and abdominal pain, 16 (76%) developed myocarditis, 17 (81%) were admitted to an intensive care unit, and 11 (52%) required mechanical ventilation; these findings suggest a pathology that is distinct from Kawasaki disease. Moreover, there have been many reports of MIS-C from Europe and the United States (11), as well as reports of disease among individuals of Afro-Caribbean and Hispanic ethnicities (4, 5). To the best of our knowledge, reports from Asia are rare, with only one report from India (12) and three reports from Korea (13).

MIS-C and Kawasaki disease are both vasculitic syndromes that are presumed to arise from cytokine storms; analyses of their cytokine characteristics may aid in understanding the underlying mechanisms of disease. Thus far, some studies have described acute phase cytokine assay findings of MIS-C (14–21), as well as differences in acute phase cytokine findings in patients with Kawasaki disease, macrophage activating syndrome, severe COVID-19, and MIS-C (15–17, 20, 21); however, we have found only a few reports that describe temporal changes in cytokines in the context of clinical symptomology for an individual patient with MIS-C. In the present report, we closely evaluated the kinetics of multiple cytokines from our patient's sera, which had been obtained prior to diagnosis and over the entire treatment trajectory.

We observed kinetics of various cytokines that are reportedly increased in patients with MIS-C, including IL-1Ra (14, 21), IL-1β (19), IL-6 (15–20, 22), IL-8 (18, 19), IL-10 (16, 20), IL-17 (17), IL-18 (20), CXCL10 (15, 17, 21, 22), tumor necrosis factor-α (18, 19), interferon-γ (20, 21), CCL3 (17), CCL20 (17), CCL19 (17), CCL2 (21, 22), CXCL1 (17), and CXCL11 (17). We found five patterns in the cytokine kinetics in contrast with patient's symptoms. Although it is not possible to clearly divide all 71 cytokines into five patterns with only a single case study, here, we demonstrate the 29 cytokines that show the most characteristic features (Figure 3). Cytokines with titers that peaked prior to the fifth day of illness (i.e., when the treatment was started; Figure 3) may be involved in the pathogenesis of MIS-C.

In particular, those shown in Figure 3A peaked out before the start of treatment, and we could observe them in daily samples obtained from the 2nd to 5th day of illness. Notably, IL-2 is associated with the *inositol-trisphosphate 3-kinase C* (*ITPKC*) gene, which was associated with susceptibility to Kawasaki disease in a previous genome-wide association study (23). It has been reported that patients with single-nucleotide polymorphisms that convey susceptibility to Kawasaki disease have reduced expression of *ITPKC*, which leads to T-cell activation and IL-2 elevation (23). We also observed the early peak of CXCL1 and CXCL2 (Figure 3A), which may suggest “neutrophil activation” involvement in the pathogenesis of MIS-C.

The cytokines with titers that peaked on the 5th day of illness and rapidly decreased after treatment as symptoms improved on the next day of treatment are shown in Figure 3B; those cytokines sensitively reflected the patient's clinical course, and thus, may be directly related to MIS-C disease activity. IL-6 is a major pro-inflammatory cytokine that was reportedly elevated in patients with MIS-C in previous studies (15–20, 22). In our patient, IL-6 titer also rapidly increased (21.1-fold) with the onset of symptoms. However, another report indicated that IL-6 elevation occurs less frequently compared with IL-1 receptor antagonist elevation (14); therefore, further data are needed regarding patients with MIS-C. The importance of IL-17 in MIS-C is controversial. Consiglio et al. report that IL-17A drives Kawasaki disease but not MIS-C hyperinflammation (15), while reports by Esteve-Sole et al. (21) and Gruber et al. (17) suggest that IL-17 is elevated in MIS-C patients. In our case, IL-17 was rapidly elevated (5.2-fold) by the time of diagnosis and decreased to less than the measurement sensitivity with fever resolution on the day after treatment initiation.

The clinical significance of cytokines with titers that did not immediately decline to baseline after the fifth day of illness (but showed a gradual decline; Figure 3C) is difficult to determine on the basis of data from our single patient; however, Caldarale et al. showed that IL-6, CXCL8, CCL2, CXCL9, and CXCL10 were higher in MIS-C patients than in pediatric COVID-19 patients, and that treatment with IVIG and methylprednisolone resulted in a rapid decrease in IL-6, but the decrease of CCL 2, CXCL9, and CXCL10 were delayed (22). The same changes in these cytokines/chemokines were observed in our study. In our case, IL-6 decreased on the day after treatment; in contrast, CXCL9, CXCL10, MCP-1 and some others took about 5 days. Caldarale et al. discussed that these interferon-γ induced chemokines (CXCL9 and CXCL10) showed significant changes in MIS-C patients and are markers of Th1 type immune response (22). In our study, in addition to CXCL9 and CXCL10, interferon-γ also showed the same pattern. The increase in IL-1 receptor antagonist titer was consistent with the findings in a previous report (14). IL-1β and IL-18 titers were only mildly elevated, and the mild elevation of the IL-18 titer was also consistent with the previously reported findings (20). These IL-1 family cytokines are reportedly associated with Kawasaki disease-related myocarditis (24), but further studies are needed to determine their clinical significance in patients with MIS-C.

Previous reports have indicated that the cytokine profiles of patients with MIS-C had some overlap and some differences from those of patients with COVID-19, macrophage activation syndrome, and Kawasaki disease (15, 16, 20, 21). Immunoglobulins and steroids have been used as treatments for these conditions and, within the past 10 years, molecular targeted therapies associated with specific cytokines (e.g., tocilizumab, infliximab, and anakinra) have become available as a treatment option. However, there remains a lack of clear evidence regarding which drugs to use for specific conditions. The identification of cytokines associated with clinical features and outcomes is necessary in understanding pathogenesis and guiding treatment decisions.

The primary limitation of this report is its focus on a single patient. Thus, similar assessments of cytokine kinetics are needed in patients with MIS-C; in particular, such reports should focus on patients who are unresponsive to treatment and on patients who receive other therapeutic agents. The findings may aid in elucidating cytokine kinetics that influence treatment responsiveness; they may also facilitate the selection of agents for add-on therapy in patients who are unresponsive to initial treatment.

## Conclusion

We conducted comprehensive temporal analyses of serum cytokine kinetics for the entire course of disease, along with an investigation of clinical symptoms, in a Japanese patient with MIS-C who exhibited a classical disease trajectory. Our findings regarding cytokines that changed during the course of disease may provide useful information for elucidating disease status and selecting therapy; the findings will be strengthened by additional analyses of cytokine kinetics in patients with MIS-C.

## Data Availability Statement

The original contributions presented in the study are included in the article/Supplementary Material, further inquiries can be directed to the corresponding author.

## Ethics Statement

The studies involving human participants were reviewed and approved by Research Ethics Committees of the National Center for Global Health and Medicine. Written informed consent to participate in this study was provided by the participants' legal guardian/next of kin.

## Author Contributions

ST and AS contributed equally to the study design and writing of the manuscript. ST, AS, and AMi designed the study and wrote the initial draft of the manuscript. AS, MS, and MM contributed to the analysis and interpretation of cytokines, and assisted in the preparation of the manuscript. YI, TM-A, AMa, and MU contributed to the analysis and interpretation of viral PCR and serum antibodies to SARS-CoV-2, and assisted in the preparation of the manuscript. HH and HS contributed to critically review of the manuscript. All authors approved the final version of the manuscript, and agree to be accountable for all aspects of the work in ensuring that questions related to the accuracy or integrity of any part of the work are appropriately investigated and resolved.

## Conflict of Interest

The authors declare that the research was conducted in the absence of any commercial or financial relationships that could be construed as a potential conflict of interest.
