# Supplementary figures and images for: Case Report: Changes in Cytokine Kinetics During the Course of Disease in a Japanese Patient With Multisystem Inflammatory Syndrome in Children
Source: Front Pediatr. 2021 Jul 21;9:702318. doi: 10.3389/fped.2021.702318 (PMC8335158; doi:10.3389/fped.2021.702318)

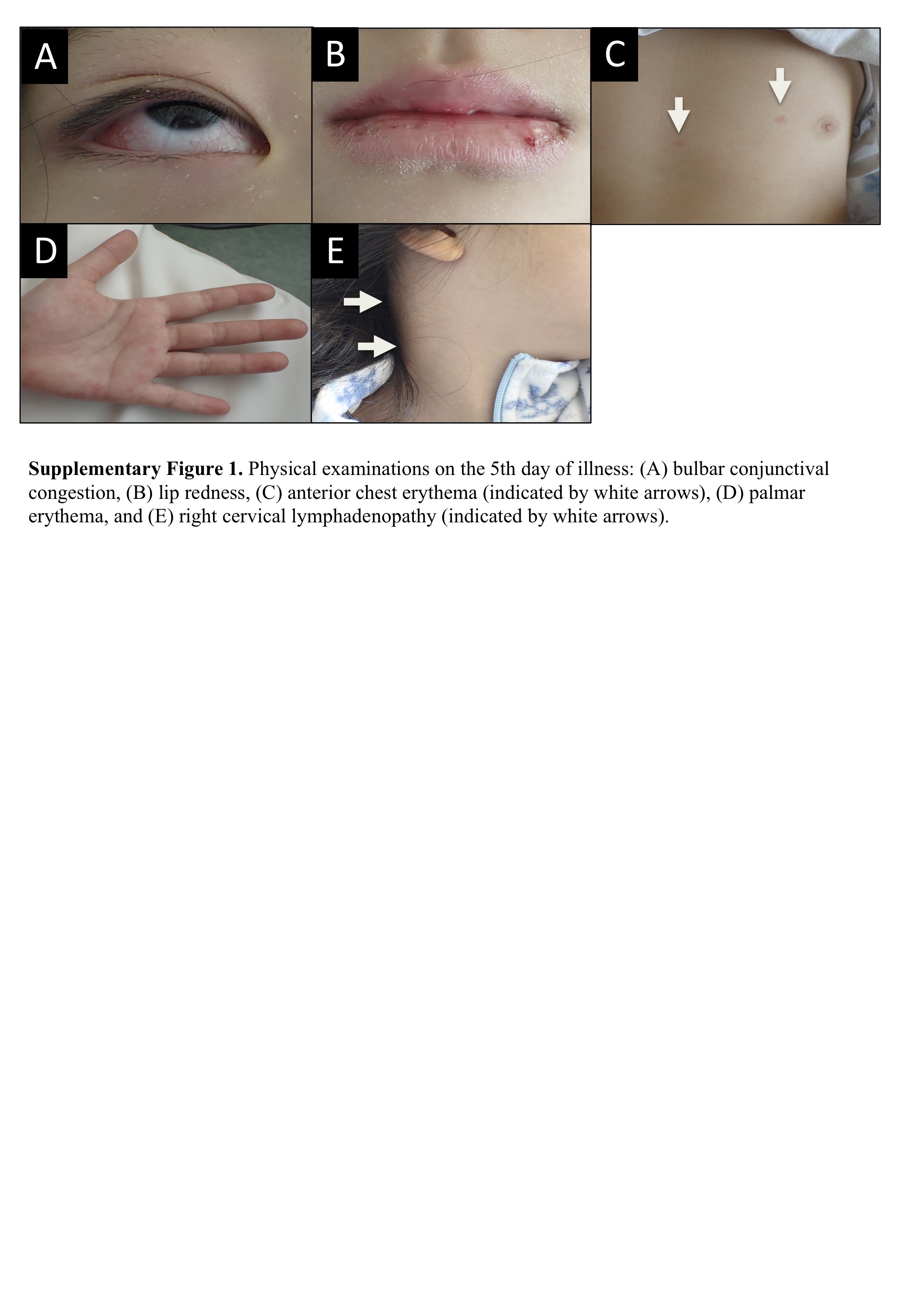

Supplement: Supplementary file 1 [file Image_1.jpg]

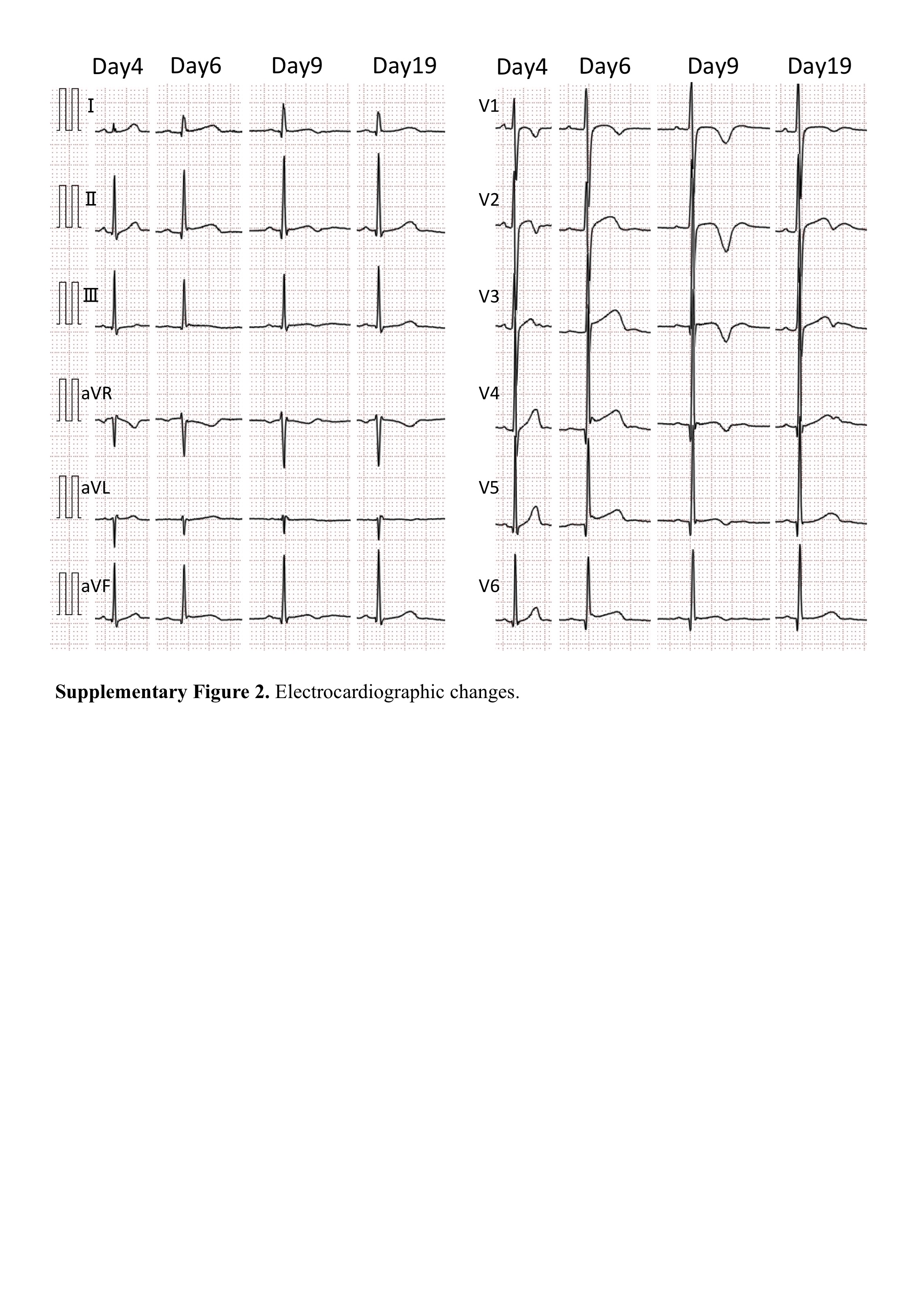

Supplement: Supplementary file 2 [file Image_2.jpg]

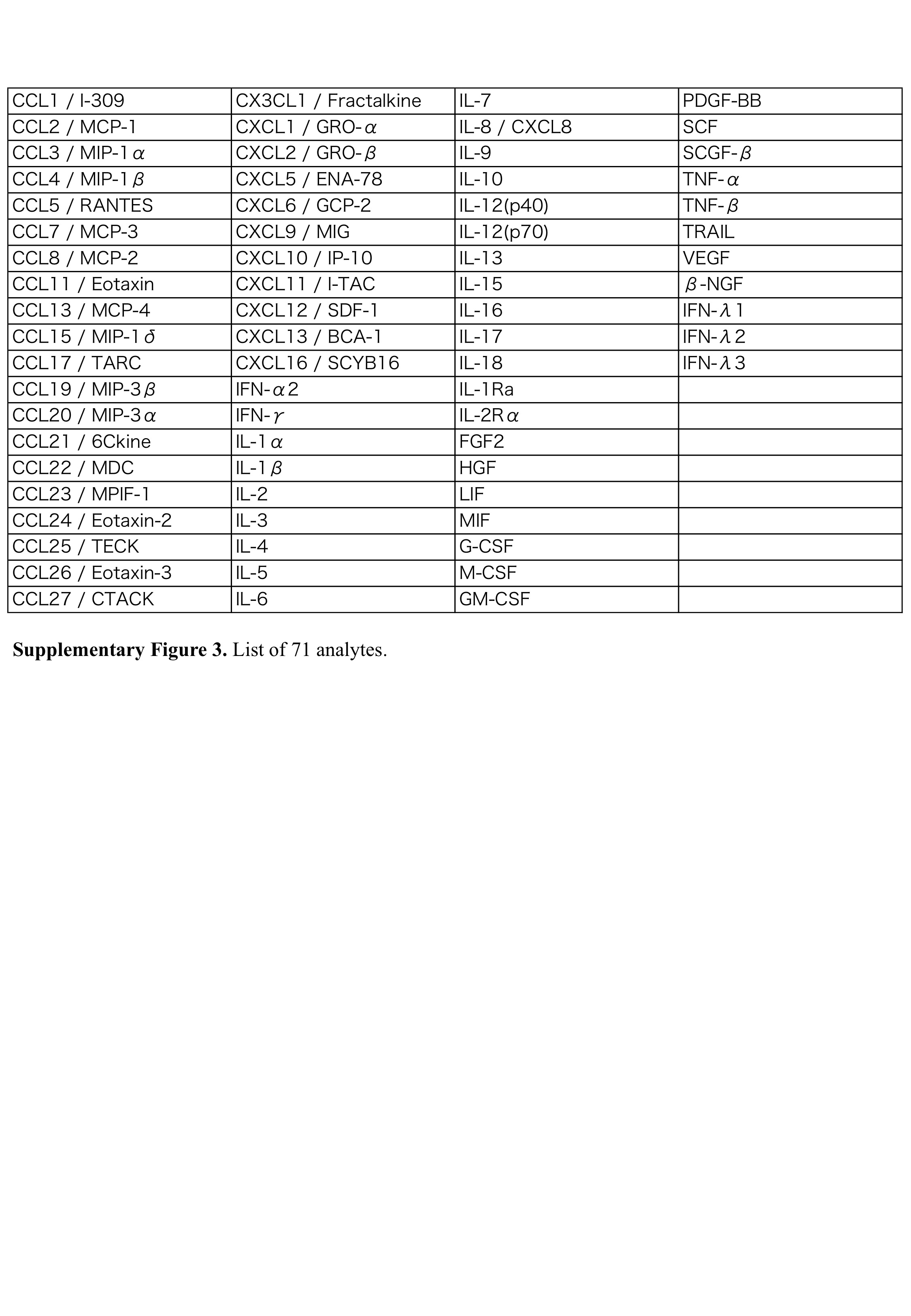

Supplement: Supplementary file 3 [file Image_3.jpg]
